# Effects of Green Kiwifruit Peel Extract on Sleep-Wake Profiles in Mice: A Polysomnographic Study Based on Electroencephalogram and Electromyogram Recordings

**DOI:** 10.3390/nu14224732

**Published:** 2022-11-09

**Authors:** Duhyeon Kim, Minseok Yoon, Seonghui Kim, Min Young Um, Suengmok Cho

**Affiliations:** 1 Department of Food Science and Technology, Institute of Food Science, Pukyong National University, Busan 48513, Korea; 2 Research Division of Food Functionality, Korea Food Research Institute, Wanju-gun 55365, Korea

**Keywords:** *Actinidia deliciosa*, electroencephalogram, green kiwifruit peel, polysomnography, sleep-promoting effect

## Abstract

In the previous study, it was reported that green kiwifruit peel ethanol extract (GKPEE) increases sleep duration and decreases sleep latency in pentobarbital-treated mice. The pentobarbital-induced sleep test can be used to verify sleep quantity, which includes factors such as sleep duration and latency, but not sleep quality. In the present study, the sleep-promoting effects of GKPEE were investigated by the analysis of electroencephalogram (EEG) and electromyogram in mice and were compared with the results of diazepam (DZP), a representative sedative-hypnotic agent. The acute administration of GKPEE (250, 500 and 1000 mg/kg) increased the amount of non-rapid eye movement sleep (NREMS) and decreased sleep latency in a dose-dependent manner. The effect of GKPEE at 1000 mg/kg produced persistently significantly different results until the second hour of time-course changes. In particular, GKPEE did not produce any change in delta activity compared to DZP. Furthermore, sub-chronic administration (15 days) of GKPEE (500 mg/kg) continued sleep-promoting effects, whilst the EEG power density of NREMS did not show significant differences, indicating that there were no tolerance phenomena. Our findings suggest that GKPEE may be a promising natural sleep aid for treating sleep disorders. In addition, considering the number of by-products discarded each year by the food industry, the application of GKPEE here contributes to the utilization of processed kiwifruit by-products and can help to solve environmental problems.

## 1. Introduction

Kiwifruit, often shortened to kiwi, is one of the most consumed and processed fruits worldwide because of its sensory properties and health benefits [1,2,3]. Kiwifruit, which belongs to the genus *Actinidia*, is cultivated in the tropics and subtropics, including New Zealand, Italy, Japan, and France [4]. It has a variety of health benefits, including antioxidant [5], anti-inflammatory [6], cardiovascular protective [7], and anti-allergic [8] effects. Studies on the health benefits of kiwifruit have led to its reputation as a superfood [9]. Moreover, flavonoids, phenolic acids, anthocyanins, tocopherols, and organic acids are considered to be the bioactive compounds responsible for its biological properties [2].

Insomnia, the most common sleep disorder, has become a widespread health complaint and a disruptive problem in modern society [10,11]. The National Sleep Foundation in the USA reported that over 80% of adults suffer from various sleep disorders at least several days per week [12]. For this reason, natural sleep aids or dietary supplements with sleep-promoting effects have been studied extensively.

Kiwifruit has also been subjected to research on the sleep-promoting effects of food. Consumption of two green kiwifruits (*Actinidia deliciosa*, 100 g each) 1 h before bedtime over a four-week intervention period improved total sleep time (by 16%), sleep efficiency (by 2.4%), and decreased sleep latency (by 14 min) and wake after sleep onset (by 6 min) in adult humans [13]. In another randomized controlled trial [14], 74 students with chronic insomnia ingested 130 g of kiwifruit 1 h before bedtime every day for four weeks. The effects of kiwifruit administration were evaluated using actigraphy, sleep diaries, and the Pittsburgh sleep questionnaire index. In this clinical study, the authors reported that kiwifruits may possess sleep-improving properties. Yang et al. [15] evaluated the hypnotic effects of green kiwifruit peel ethanol extract (GKPEE) in mice using the pentobarbital-induced sleep test. GKPEE (250–1000 mg/kg) exhibited a dose-dependent increase in sleep duration and decrease in sleep latency in mice treated with pentobarbital.

Although the hypnotic effects of kiwifruit and its extract have been studied in animal assays and clinical trials, the effects of kiwifruit on sleep-wake profiles in rodents based on electroencephalogram (EEG) and electromyogram (EMG) have not yet been demonstrated. The analysis of sleep architecture using polysomnographic recordings has been considered the most important assay for evaluating sleep-promoting effects [16]. To the best of our knowledge, this is the first study to evaluate the sleep-promoting effects of GKPEE by recording and analyzing EEG and EMG in mice. In the present study, we investigated the changes in rapid eye movement sleep (REMS), non-REMS (NREMS), sleep latency, and delta activity in mice following the acute and sub-chronic administration (15 days) of GKPEE.

## 2. Materials and Methods

### 2.1. Materials

A barbiturate anesthetic pentobarbital was purchased from Hanlim Pharm. Co., Ltd. (Seoul, Korea) for surgery on mice. Diazepam (DZP) was purchased from Myungin Pharm. Co., Ltd. (Seoul, Korea) and used as a reference sedative-hypnotic drug. All the chemicals and reagents used in this study were of analytical grade.

### 2.2. Preparation of GKPEE

Green kiwifruit (ZESPRI^®^, weight range: 100–110 g) was purchased from a local market in Korea. Peels from green kiwifruits were extracted with a 95% (*v/v*) ethanol-water solution at 50 °C for three days. The ethanol extraction solution was then concentrated, freeze-dried, and powdered. The yield of GKPEE was 7.9% (*w/w*).

### 2.3. Animals

For the analysis of sleep structure, C57BL/6N mice (male) weighing 27–30 g were used. The mice were obtained from Koatech Animal, Inc. (Pyeongtaek, Korea). Animals were housed with ad libitum access to food and water. The cages were maintained under controlled lighting (12 h light/dark cycle), temperature (24 °C), and humidity (55%). The experimental procedures involving animals were conducted in accordance with the animal care and use guidelines of the Korea Food Research Institutional Animal Care and Use Committee (permission number: KFRI-M-19012) and the Institutional Animal Care and Use Committee of Pukyong National University (permission number: PKNUIACUC-2021-43).

### 2.4. Analysis of Sleep Architecture

#### 2.4.1. Acute Administration

For oral administration, GKPEE and DZP were suspended in 0.5% (*w/v*) carboxymethyl cellulose-physiological saline immediately before use. GKPEE (250, 500, and 1000 mg/kg/day) and DZP (2 mg/kg/day) were administered via oral gavage to five groups of C57BL/6N mice (n = 7–8 per group) at 09:00 AM on the day of the experiment.

#### 2.4.2. Sub-Chronic Administration

**Figure 1:**
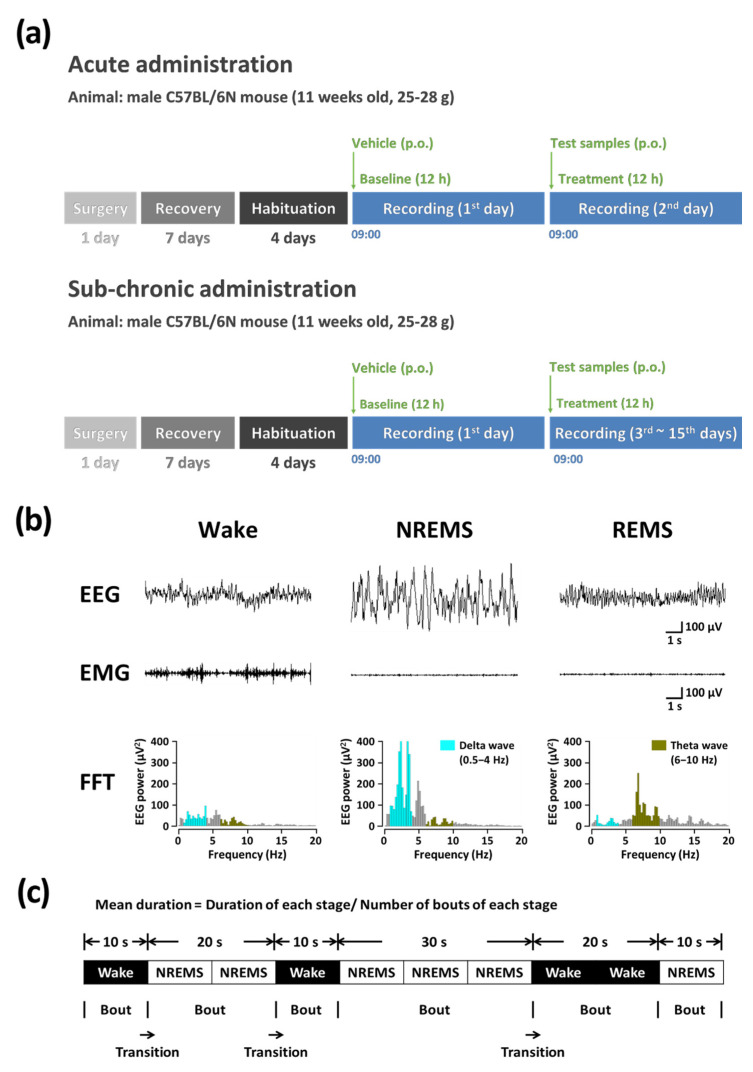
(**a**) Experimental procedure of acute and sub-chronic (15 days) administration for polysomnographic recordings. (**b**) Typical EEG, EMG, and FFT spectra in C57BL/6N mice. (**c**) Definition of sleep-wake episodes. EEG, electroencephalogram; EMG, electromyogram; FFT, fast Fourier transform; NREMS, non-rapid eye movement sleep; p.o., per os injection; REMS, rapid eye movement sleep; Wake, wakefulness.

The experimental procedure for the sub-chronic administration is shown in Figure 1a. The GKPEE (500 mg/kg) was administered orally (p.o.) to C57BL/6N mice (n = 7–8 per group) daily at 09:00 AM. The administration lasted for 15 consecutive days, and EEG and EMG were recorded on the 1st (baseline), 3rd, 6th, 9th, 12th, and 15th days.

#### 2.4.3. Polysomnographic Recordings and Vigilance State

A head mount (#8201; Pinnacle Technology, Inc., Lawrence, KS, USA) consisting of EEG and EMG electrodes was inserted into the skulls of the mice to collect polysomnographic signals. To anesthetize C57BL/6N mice, the pentobarbital (50 mg/kg) was injected through intraperitoneal injection (Figure 1b). The mice were cleaned with 70% ethanol before surgery, and the mice’s hair was removed from head to neck. The front edge of the head mount was inset anterior to the bregma. Subsequently, two 0.1-inch screws were inserted into the front holes, and two 0.12-inch screws were inserted into the back holes. The two EMG wire electrodes penetrated small pockets made in the nuchal muscles. Finally, the dental cement was covered to fix the head mount onto the skull. Subsequently, the mice were separated into individual cages for one week. After recovery, the mice were habituated under recording conditions for four days. The polysomnographic recording signals were collected using a PAL-8200 data acquisition system (Pinnacle Technology Inc., Lawrence, KS, USA). The EEG and EMG signals were amplified 100-fold and low-pass filtered at 25 Hz. All signals were stored at a sampling rate of 200 Hz. Recording started at 09:00 AM, immediately after oral administration of the samples, and was conducted for 12 h. The sleep-wake stages were classified by a 10 s epoch as NREMS, rapid eye movement, and wake using SleepSign (version 3.0; Kissei Comtec, Nagano, Japan). We measured sleep latency as the time lasting more than 2 min between sample administration and the first consecutive NREMS. Delta activity ranging from 0.5 to 4 Hz was summated and normalized to obtain the ratio of the corresponding average delta power during NREMS. Delta activity has been used as an indicator of the depth of sleep as well as the quality of sleep [17]. Bouts of sleep-wake stages (wake, NREMS, and REMS) were defined as periods of one or more consecutive 10 sec epochs (Figure 1c).

### 2.5. Statistical Analysis

The data are presented as mean ± standard error of the mean (SEM). The statistical analysis was assessed using Prism (version 8.0; GraphPad Software Inc., San Diego, CA, USA).

In order to compare them, the two groups of data were analyzed by paired Student’s *t*-test. For multiple comparisons in the pentobarbital-induced sleep test, the data were assessed using a one-way analysis of variance (ANOVA) followed by Dunnett’s test. The significance level was set at *p* < 0.05 for all statistical tests.

## 3. Results

### 3.1. Effects of Acute Administration of GKPEE on Sleep-Wake Profiles

**Figure 2:**
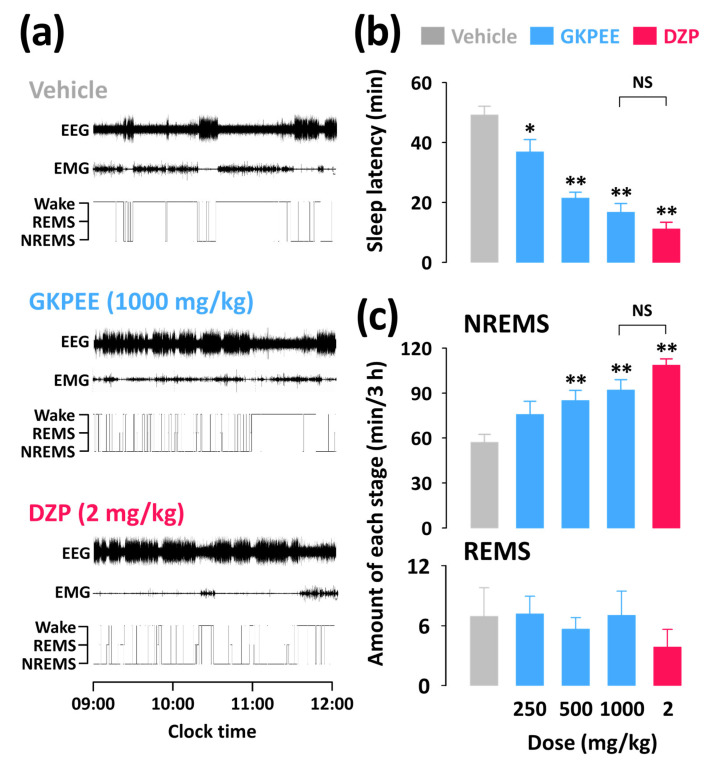
Effects of GKPEE and DZP on sleep profiles in mice. (**a**) Examples of EEG/EMG signals and hypnograms in a mouse treated with GKPEE and DZP. (**b**) Changes in sleep latency by administration of GKPEE and DZP. (**c**) NREMS and REMS amounts during the 3 h period following injection of GKPEE and DZP. Columns represent the mean ± SEM of 7–8 mice. * *p* < 0.05 and ** *p* < 0.01, are significant compared to the vehicle (Dunnett’s test). DZP, diazepam; EEG, electroencephalogram; EMG, electromyogram; GKPEE, green kiwifruit peel ethanol extract; NS, no significance; REMS, rapid eye movement sleep; NREMS, non-REMS; SEM, standard error of mean; Wake, wakefulness.

To better understand the sleep-promoting effects of GKPEE on normal sleep in mice, we analyzed sleep profiles based on polysomnographic recordings. Examples of polysomnographic recordings and hypnograms from a single mouse during the first 3 h after the injection of the vehicle, GKPEE, or DZP are presented in Figure 2a. Oral administration of GKPEE (250–1000 mg/kg) decreased sleep latency dose-dependently (Figure 2b). The values of sleep latency were 16.8 ± 2.9 min and 11.1 ± 2.2 min in C57BL/6N mice treated with GKPEE (1000 mg/kg) and DZP (2 mg/kg), respectively. We then determined the amounts of NREMS and REMS during the first 3 h after the administration of GKPEE or DZP (Figure 2c). The administration of GKPEE at 500 and 1000 mg/kg significantly increased the amount of NREMS by 1.5- and 1.6-fold, respectively, compared to the vehicle group (*p* < 0.01). DZP also significantly increased NREMS by 1.9-fold (*p* < 0.01) compared with the vehicle. No Significant differences in the NREMS amount between GKPEE (1000 mg/kg) and DZP (2 mg/kg) were observed. In addition, the amount of REMS produced by GKPEE and DZP was not significantly different.

### 3.2. Effects of Acute Administration of GKPEE on Time-Course Changes in Each Sleep Stage

**Figure 3:**
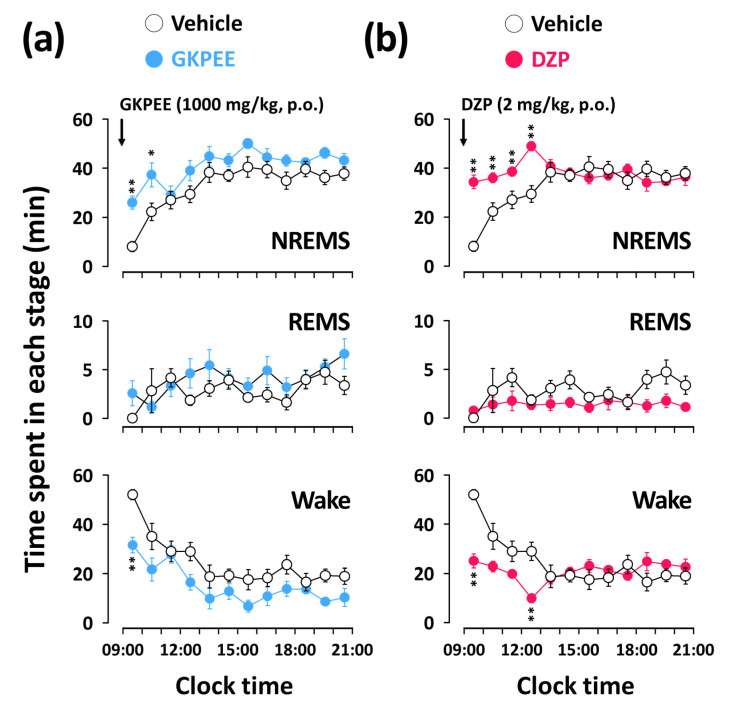
Effects of GKPEE (**a**) and DZP (**b**) on time-course changes in amounts of NREMS, REMS, and Wake during 12 h in mice. Open circle indicates the vehicle and blue and red filled circles represent GKPEE and DZP, respectively. * *p* < 0.05 and ** *p* < 0.01 were considered significant as compared to the vehicle (paired Student’s t-test). DZP, diazepam; GKPEE, green kiwifruit peel ethanol extract; REMS, rapid eye movement sleep; NREMS, non-REMS; SEM, standard error of the mean; Wake, wakefulness.

Figure 3 shows the time-course changes in each sleep stage for 12 h after the administration of GKPEE (1000 mg/kg) and DZP (2 mg/kg). GKPEE at 1000 mg/kg significantly increased the NREMS amount during the 2 h following injection by 3.2- (*p* < 0.01) and 1.7-fold (*p* < 0.05), respectively, relative to the vehicle (Figure 3a). This enhancement of NREMS was accompanied by the reduction of wakefulness. During subsequent periods, no further significant disruption was observed in any sleep or wake stages. The DZP effects lasted for 4 h after administration (Figure 3b). Neither GKPEE nor DZP caused any significant difference in the amount of REMS in the 12 h after administration.

### 3.3. Effects of Acute Administration of GKPEE on Sleep-Wake Episode and Delta Activity

**Figure 4:**
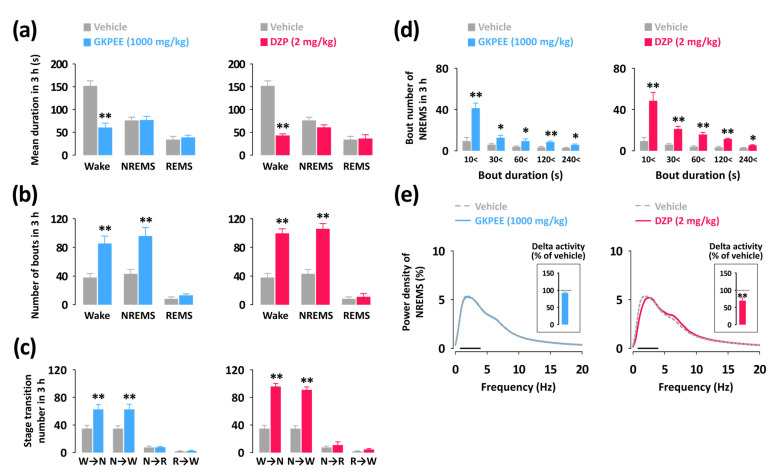
Effects of GKPEE and DZP on sleep-wake episodes in mice during the 3 h period after injection. (**a**) Mean duration and (**b**) total number of Wake, NREMS, and REMS bouts in a 3 h period after the administration of GKPEE and DZP. (**c**) Sleep-wake stage transitions during the 3 h following administration of GKPEE and DZP. (**d**) Changes in the number of NREMS bouts of different bout duration in C57BL/6N mice following administration of GKPEE or DZP. Columns represent the mean ± SEM of 7–8 mice. (**e**) EEG power density curves of NREMS caused by GKPEE and DZP. The bar (—) represents the range of the delta wave (0.5–4 Hz). * *p* < 0.05, ** *p* < 0.01 were considered significant as compared to the vehicle (paired Student’s t-test). DZP, diazepam; GKPEE, green kiwifruit peel ethanol extract; NS, no significance; REMS (or R), rapid eye movement sleep; NREMS (or N), non-REMS; SEM, standard error of mean; Wake (or W), wakefulness.

Subsequently, changes in the mean duration and characteristics of sleep-wake episodes by GKPEE and DZP were analyzed (Figure 4). Both DZP and GKPEE showed a significant decrease in the mean duration of wake episodes (GKPEE: 60%, *p* < 0.05; DZP: 72%, *p* < 0.05) without changing the duration of NREMS of REMS (Figure 4a). In addition, GKPEE increased the number of wake and NREMS bouts 2.3- and 2.6-fold, respectively (Figure 4b). The number of transitions from Wake to NREMS (W→N) and N→W significantly increased after the administration of GKPEE or DZP (Figure 4c). GKPEE significantly elevated the number of bouts of NREMS in all ranges in the 3 h after administration compared to the vehicle (Figure 4d).

To evaluate sleep intensity, we calculated the delta activity (frequency range, 0.5–4 Hz) in C57BL/6N mice during NREMS (Figure 4e). DZP significantly (*p* < 0.01) decreased delta activity. However, the administration of GKPEE did not affect EEG power density or delta activity.

### 3.4. Effects of Sub-Chronic Administration of GKPEE on Sleep-Wake Profiles and Delta Activity

**Figure 5:**
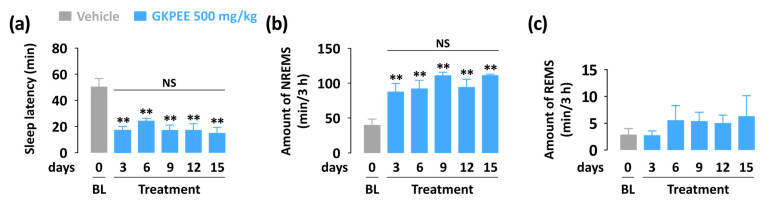
Effect of sub-chronic (15 days) administration of GKPEE on sleep latency (**a**). Amounts of NREMS (**b**) and REMS (C) during the 3 h after administration. (**c**) REMS amount. Columns represent the mean ± SEM of 7–8 mice. ** *p* < 0.01, significant as compared to the vehicle (Dunnett’s test). BL, baseline; GKPEE, green kiwifruit peel ethanol extract; NS, no significance; REMS, rapid eye movement sleep; NREMS, non-REMS; SEM, standard error of mean.

Based on the above results, we confirmed that GKPEE promotes NREMS after acute administration. Additionally, we performed a sub-chronic (15-day) administration test to identify whether GKPEE has sleep-promoting effects. Sleep latency was persistently reduced during the 15 days of GKPEE administration (Figure 5a). NREMS and REMS amounts during the 3 h after GKPEE treatment were calculated. As expected, GKPEE produced a significant increase in the amount of NREMS throughout the 15 days (Figure 5b). However, changes in the amount of REMS did not occur (Figure 5c).

**Figure 6:**
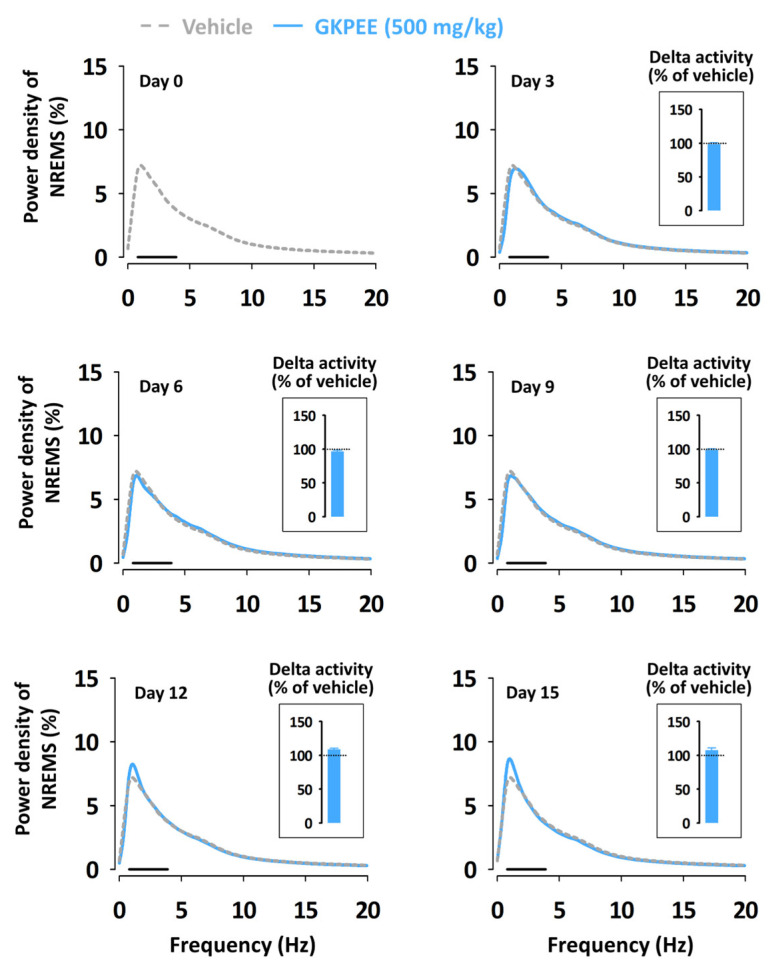
Effect of Sub-chronic (15-day) administration of GKPEE on EEG power density curves of NREMS. The bar (—) represents the range of the delta wave (0.5–4 Hz). GKPEE, green kiwifruit peel ethanol extract; NREMS, non-rapid eye movement sleep.

To identify whether the sleep-promoting effects of GKPEE persist throughout the sub-chronic (15-day) treatment period, the delta activity of GKPEE was measured (Figure 6). The delta activity of GKPEE did not change throughout the administration period.

## 4. Discussion

In the previous investigation, the hypnotic effects of GKPEE in animals were demonstrated using the pentobarbital-induced sleep test [15]. The oral administration (500 and 1000 mg/kg) of GKPEE resulted in a significant increase in sleep duration and decrease in sleep latency in imprinting control region mice. As shown in Table 1, the pentobarbital-induced sleep test is the most common method for screening compounds with suspected sedative-hypnotic effects [18,19]. However, it has the limitation that the hypnotic effects may be exhibited even when the sample has toxic or adverse effects [20]. In addition, this method can evaluate sleep quantity, such as sleep duration and latency, but not sleep quality [21]. Taking into consideration the limitations of the pentobarbital-induced sleep test, the hypnotic effects of GKPEE in mice treated with pentobarbital do not confirm that it has sleep-promoting or sleep-enhancing effects.

In this study, we analyzed sleep architecture to identify the sleep-promoting effects of GKPEE based on EEG and EMG recordings. Our findings showed that GKPEE at the dosages of 500 and 1000 mg/kg significantly decreased sleep latency and increased NREMS (Figure 2b,c) during the first 3 h after injection. A previous study revealed that DZP, a positive allosteric modulator of the GABA_A_-benzodiazepine (BZD) receptor, potentiates NREMS in mice [22]. The current findings clearly show that DZP also produced an elevated level of NREMS without affecting REMS (Figure 2c). The time-course changes in NREMS after the administration of GKPEE were less than that of DZP but increased during the second hour (Figure 3). GKPEE not only significantly increased NREMS bouts but also increased the number of stage transitions from W→N and N→W (Figure 4). Our data suggest that GKPEE induces NREMS similarly to DZP. EEG delta (0.5–4.0 Hz) activity in NREMS is a well-known indicator of sleep [23]. Sedative-hypnotic drugs, such as zolpidem and DZP, have been reported to inhibit delta activity, affecting the depth and intensity of sleep [24]. In the present study, we confirmed that DZP reduces delta activity. A previous study reported that the sleep-promoting effects of GKPEE were significantly inhibited by the GABA_A_-BZD antagonist, flumazenil [15]. This finding implies that GKPEE exhibits hypnotic effects via the modulation of the BZD-binding site of GABA_A_ receptors, as DZP does. However, GKPEE appears to act as a GABA_A_-BZD agonist, similar to DZP, promoting NREMS without significantly reducing delta activity.

No reduction in the sleep-promoting effects of GKPEE was observed in the sub-chronic (15 days) administration test (Figure 5). The EEG power density of NREMS showed no significant difference between the GKPEE treatment and the vehicle during the entire administration period (Figure 6). This result implies that GKPEE has no tolerance issues with long-term administration. It is widely known that chronic treatment with zolpidem or DZP does not have hypnotic effects in animal models [25,26]. DZP and zolpidem cause many side effects, such as memory and cognitive impairment [27]. They induce tolerance phenomena and dependence during chronic administration [28]. It might be possible that tolerance to sedative effects is mediated by the α1 subunit and the loss of the synaptic function of the GABA_A_-BZD receptor after long-term administration [29]. Thus, it appears that GKPEE does not result in the loss of synaptic functions in the GABA_A_-BZD receptor, or that improves sleep-promoting effects through the activation of another subunit. The sleep-promoting effects of GKPEE, which does not cause tolerance, may be beneficial as a natural sleep aid.

In this study, since fruits generally contain more flavonoids in the peel than in the pulp, we conducted experiments using kiwifruit peel extract [30,31]. Flavonoids in fruits, such as quercetin and kaempferol, help to improve the quality of sleep [32,33]. In addition, a recent study reported that the total phenolic content and total flavonoid content of the peel were higher than that of the pulp and the core [34]. Thus, it is preferential to use the peel, which contains more of the bioactive compound, than any other parts of the kiwifruit.

## 5. Conclusions

In summary, our study revealed that GKPEE increases the NREMS amount and decreases sleep latency in C57BL/6N mice. We compared the in vivo hypnotic effects between DZP and GKPEE based on the results of the pentobarbital-induced sleep test and polygraphic recordings (Table 2). The significant increase in the sleep quantity (e.g., sleep duration) was observed for both DZP and GKPEE in both methods, whereas the sleep intensity (e.g., delta activity) was not observed in our polygraphic study. GKPEE did not decrease delta activity, unlike the hypnotic drug DZP, which reduced it significantly. After the comparison with the well-known dietary supplement for sleep, the recommended dose of GKPEE in the sub-chronic administration test was determined at 500 mg/kg. Therefore, considering the recommended daily intake in humans, it is desirable to consume GKPEE at 500 mg/kg. Our findings suggest that GKPEE is a promising therapeutic agent for insomnia treatment. Further studies are needed to evaluate the sleep-promoting effects of GKPEE by polysomnography in human clinical trials. On the other hand, kiwifruit peel by-products generated plentifully in industrial processing have several applications, such as juice, smoothies, sweets, and ice creams [3]. The industrial utilization of fruit by-products is a major global trend for addressing the issue of sustainability in the food industry [35]. As thousands of tons of kiwifruit by-products are discarded, it is necessary to consider them as a valuable source of health functional ingredients [3]. Therefore, the demonstration of the sleep-promoting effects of GKPEE provides an additional application that could contribute to the more efficient utilization of kiwifruit processing by-products and serve as a solution to certain economic and environmental problems.

## Figures and Tables

**Table 1:** In vivo evaluation methods for assessing hypnotic effects and demonstration of hypnotic effects of the kiwifruit peel extract.

Method	Pentobarbital-Induced Sleep Test		Polygraphic Recordings	
Animal	ICR mouse		C57BL/6N mouse	
Measurements	Righting reflex		EEG and EMG	
Advantages	Short assay time, possible to screen many samples		Assessment of both sleep quantity and quality	
Disadvantages	Impossible to evaluate sleep quality		Long assay time, high cost	
Evaluation markers and hypnotic effects of the kiwifruit peel extract	Sleep latency Sleep duration	Significant decrease [15] Significant increase [15]	Sleep latency NREMS amount REMS amount Delta activity	None None None None

EEG, electroencephalogram; EMG, electromyogram; ICR, imprinting control region.

**Table 2:** Comparison of effectiveness between DZP and GKPEE.

Method	Pentobarbital-Induced Sleep Test		Polygraphic Recordings	
Hypnotic agents	DZP	GKPEE	DZP	GKPEE
Sleep quantity	Increase	Increase	Increase	Increase
Sleep intensity	None	None	Decrease	No effect

DZP, diazepam; GKPEE, green kiwifruit peel ethanol extract.

## Data Availability

Not applicable.
